# Does primary neoadjuvant systemic therapy eradicate minimal residual disease? Analysis of disseminated and circulating tumor cells before and after therapy

**DOI:** 10.1186/s13058-016-0679-3

**Published:** 2016-02-12

**Authors:** Sabine Kasimir-Bauer, Ann-Kathrin Bittner, Lisa König, Katharina Reiter, Thomas Keller, Rainer Kimmig, Oliver Hoffmann

**Affiliations:** Department of Gynecology and Obstetrics, University Hospital Essen, University of Duisburg-Essen, Hufelandstrasse 55, D-45122 Essen, Germany; ACOMED Statistik, Fockestrasse 57, D-04275 Leipzig, Germany

**Keywords:** Breast cancer, Circulating tumor cells, Disseminated tumor cells, Minimal residual disease neoadjuvant therapy, Stem cells, EMT

## Abstract

**Background:**

Patients with breast cancer (BC) undergoing neoadjuvant chemotherapy (NACT) may experience metastatic relapse despite achieving a pathologic complete response. We analyzed patients with BC before and after NACT for disseminated tumor cells (DTCs) in the bone marrow(BM); comprehensively characterized circulating tumor cells (CTCs), including stem cell–like CTCs (slCTCs), in blood to prove the effectiveness of treatment on these cells; and correlated these findings with response to therapy, progression-free survival (PFS), and overall survival (OS).

**Methods:**

CTCs (*n* = 135) and slCTCs (*n* = 91) before and after NACT were analyzed using the AdnaTest BreastCancer, AdnaTest TumorStemCell, and epithelial–mesenchymal transition (QIAGEN Hannover GmbH Germany). The expression of estrogen receptor, progesterone receptor, and the resistance marker excision repair cross-complementing rodent repair deficiency, complementation group 1 (ERCC1), nuclease were studied in separate single-plex reverse transcription polymerase chain reaction experiments. DTCs were evaluated in 142 patients before and 165 patients after NACT using the pan-cytokeratin antibody A45-B/B3 for immunocytochemistry.

**Results:**

The positivity rates for DTCs, CTCs, and slCTCs were 27 %, 24 %, and 51 % before and 20 %, 8 %, and 20 % after NACT, respectively. Interestingly, 72 % of CTCs present after therapy were positive for ERCC1, and CTCs before (*p* = 0.005) and after NACT (*p* = 0.05) were significantly associated with the presence of slCTCs. Whereas no significant associations with clinical parameters were found for CTCs and slCTCs, DTCs were significantly associated with nodal status (*p* = 0.03) and histology (0.046) before NACT and with the immunohistochemical subtype (*p* = 0.02) after NACT. Univariable Cox regression analysis revealed that age (*p* = 0.0065), tumor size before NACT (*p* = 0.0473), nodal status after NACT (*p* = 0.0137), and response to NACT (*p* = 0.0136) were significantly correlated with PFS, whereas age (*p* = 0.0162) and nodal status after NACT (*p* = 0.0243) were significantly associated with OS. No significant correlations were found for DTCs or any CTCs before and after therapy with regard to PFS and OS.

**Conclusions:**

Although CTCs were eradicated more effectively than DTCs, CTCs detected after treatment seemed to be associated with tumor cells showing tumor stem cell characteristics as well as with resistant tumor cell populations that might indicate a worse outcome in the future. Thus, these patients might benefit from additional second-line treatment protocols including bisphosphonates for the eradication of DTCs.

**Electronic supplementary material:**

The online version of this article (doi:10.1186/s13058-016-0679-3) contains supplementary material, which is available to authorized users.

## Background

Neoadjuvant chemotherapy (NACT) was initially used to treat locally advanced as well as inoperable tumors and now has become a standard treatment in primary breast cancer (BC). Because no difference between NACT and adjuvant treatment in terms of overall survival (OS) and the probability of disease relapse [progression-free survival (PFS)] has been demonstrated, NACT is also offered to patients with resectable tumors [1, 2]. Thus, for most patients affected with primary BC, standard care is now NACT followed by surgical resection of the malignant tissue, offering the possibility of monitoring primary tumor response to treatment [3]. The surgical procedure might be influenced by tumor regression due to NACT. The evaluation of the effect of NACT is based on the assessment of local tumor response measured by clinical assessment; imaging modalities such as ultrasound, mammography, or magnetic resonance imaging of the breast; and postsurgery histopathological examination [4]. The primary aim of NACT is to reduce tumor size before therapy, monitor tumor response to treatment, and eradicate micrometastases.

However, although a pathological complete response (pCR) can be achieved in a range of 7.7–36.4 % of cases (depending on intrinsic subtypes), showing improved long-term survival, about 20 % of all patients with BC will develop metastatic relapse [5]. Relapse is often explained by early micrometastatic spread to blood, reflected by circulating tumor cells (CTCs), and to bone marrow (BM), reflected by disseminated tumor cells (DTCs) in up to 40 % of the patients [6, 7]. In this regard, the presence and persistence of CTCs and DTCs have been widely accepted as independent prognostic markers with regard to increased risk for shorter PFS and OS. Consequently, these markers have been used as a monitoring tool for adjuvant, neoadjuvant, and secondary adjuvant treatment in primary BC [8–24]. However, the detection of DTCs and CTCs has not been included in clinical routine, and therapeutic consequences according to the presence of these cells have rarely been examined. Regarding DTCs, we and others have reported that bisphosphonates, zoledronic acid, and clodronate contribute to the eradication of DTCs, even years after the first diagnosis [25–28]. Regarding CTCs, clinical studies are ongoing to evaluate targets on CTCs for additional therapeutic strategies. In this context, the DETECT III phase III trial researchers in Germany are comparing standard therapy alone with standard therapy plus human epidermal growth factor receptor 2 (HER2)-targeted therapy in patients with initially HER2-negative metastatic BC and HER2-positive CTCs [29]. In addition, the Treat CTC trial investigators are evaluating the effectiveness of trastuzumab in eliminating persisting CTCs in patients with an HER2-negative primary tumor after (neo-)adjuvant chemotherapy and surgery [30].

The reason why the detection of minimal residual disease has rarely been followed by therapeutic interventions is the broad heterogeneity of these cells, which makes therapeutic interventions difficult. Phenotyping of both cell types in primary BC has demonstrated a discordant estrogen receptor (ER), progesterone receptor (PR), and/or HER2 receptor status between the primary tumor and these cells and that a proportion of DTCs and CTCs are nonproliferative and stem cell–like as well as being in epithelial–mesenchymal transition (EMT), which may explain resistance to antihormonal and conventional chemotherapeutic drugs [31–40]. In this context, Creighton et al. found supported evidence that the residual breast tumor tissue cell populations surviving after letrozole or docetaxel treatment were enriched for subpopulations of cells with both tumor-initiating and mesenchymal features [41]. These data might also explain the results of the REMAGUS 02 neoadjuvant phase II study, which showed that although CTCs before therapy significantly correlated with PFS and OS, tumor response to chemotherapy was interestingly not correlated with CTC detection before and/or after NACT [42]. Similar results were also described in the neoadjuvant GeparQuattro Trial [43]. In a recently published meta-analysis summarizing the results of several prospective randomized trials of the change in CTC counts before and after NACT, researchers confirmed the loss of association between the decrease of CTC number and pCR [44]. Despite the prognostic impact of CTC counts, a comprehensive analysis of these cells, especially after NACT, would be challenging with regard to identifying predictive markers that might allow physicians to tailor treatment accordingly as well as to prevent exposure to ineffective therapies. In this regard, it has been demonstrated that a molecular method (AdnaTest; AdnaGen, Langenhagen Germany) can complement cell counting (CELLSEARCH; Janssen Diagnostics, Raritan, NJ, USA) in metastatic BC [45, 46]. In the neoadjuvant setting, to the best of our knowledge, no comprehensive characterization of CTCs has been performed until now.

We analyzed 190 patients with BC before and after NACT for DTCs in the BM and further characterized CTCs by molecular profiling, including HER2, the hormonal receptors ER and PR, and stem cell–like CTCs (slCTCs) [aldehyde dehydrogenase 1 (ALDH1) and/or EMT-like]. According to results obtained in our patients with metastatic BC, we additionally analyzed the resistance marker excision repair cross-complementing rodent repair deficiency, complementation group 1 (ERCC1), nuclease. It was the purpose of our study to prove the effectiveness of treatment on DTCs and CTCs and to correlate these findings with clinical parameters, response to therapy, PFS, and OS. Besides evaluating the prognostic impact of tumor cells, one of our main goals was to identify insufficiently treated patients by comprehensive molecular characterization of CTCs for possible secondary treatment options.

## Methods

### Patient population and patient characteristics

The study was conducted in the Department of Gynecology and Obstetrics at the University Hospital of Essen. In total, 190 patients with diagnosed primary, nonmetastatic BC between January 2007 and June 2012 and treated with NACT were enrolled. Patient characteristics are documented in Table 1.

**Table 1:** Patient characteristics

Characteristics	Data
Total number of patients	190
Age, yr	51 (18–84)
Menopausal status	
Premenopausal	87 (46 %)
Perimenopausal	24 (13 %)
Postmenopausal	79 (42 %)
Histologic findings	
Ductal	140 (74 %)
Lobular	22 (12 %)
Other	24 (13 %)
Unknown	4 (2 %)
Tumor grading	
G1	13 (7 %)
G2	85 (45 %)
G3	88 (46 %)
Unknown	4 (2 %)
Tumor size before NACT	
cT1a–cT1c	46 (24 %)
cT2	111 (58 %)
Above cT2	29 (15 %)
Unknown	4 (2 %)
Tumor size after NACT	
ypT0(is)	46/177 (26 %)
ypT1a–ypT1c	71/177 (40 %)
ypT2	47/177 (27 %)
Above ypT2	13/177 (7 %)
Unknown	13/190
Nodal status before NACT	
cN0	94 (49 %)
cN1	83 (44 %)
cN2, cN3	10 (5 %)
Unknown	3 (2 %)
Nodal status after NACT	
yN0	116/180 (64 %)
yN1	48/180 (27 %)
yN2,N3	16/180 (9 %)
Unknown	10/190
Estrogen receptor	
Positive	131 (69 %)
Negative	58 (31 %)
Unknown	1 (1 %)
Progesterone receptor	
Positive	116 (61 %)
Negative	73 (38 %)
Unknown	1 (1 %)
HER2 status	
Positive	56 (29 %)
Negative	133 (70 %)
Unknown	1 (1 %)
Tumor subtype (IHC)	
ER−, PR−, HER2−	36 (19 %)
ER−, PR−, HER2+	15 (8 %)
ER+/PR+, HER2−	97 (51 %)
ER+, PR+, HER2+	41 (22 %)
Unknown/n.a.	1 (1 %)
Pathological response	
Complete response	37/176 (21 %)
Partial response	127/176 (72 %)
No response	12/176 (7 %)
Unknown	14 (7 %)
DTC positive	
Before therapy	38/142 (27 %)
After therapy	33/165 (20 %)
CTC positive	
Before therapy	32/135 (24 %)
After therapy	11/133 (8 %)
slCTC positive	
Before therapy	46/91 (51 %)
After therapy	18/90 (20 %)
DTC and/or CTC positive	
Before therapy	59/136 (43 %)
After therapy	44/140 (34 %)
DTC and/or slCTC-pos.	
Before therapy	74/107 (69 %)
After therapy	48/103 (47 %)
CTC and/or slCTC positive	
Before therapy	57/92 (62 %)
After therapy	25/89 (28 %)
Survival	
OS	54 mo (2–93 mo)
Alive	169 (89 %)
Dead	19 (10 %)
Unknown	2 (1 %)
PFS	52 mo (2–93 mo)
Recurrence	
Alive without relapse	135 (71 %)
Relapse	22 (12 %)
Unknown	33 (17 %)

*CTC* circulating tumor cell, *DTC* disseminated tumor cell, *ER* estrogen receptor, *HER2* human epidermal growth factor receptor 2, *IHC* immunhistochemstry, *NACT* neoadjuvant chemotherapy, *OS* overall survival, *PFS* progression-free survival, *PR* progesterone receptor, *slCTC* stem cell–like circulating tumor cell

Data are presented as median (range) or number (%)

### Study design

We conducted a retrospective, single-institution trial to determine the prognostic value of DTCs in the BM and CTCs in blood of the patients and proved the effectiveness of treatment on these cells. The median follow-up time was 54 months (range 2–93 months) for OS and 52 months (range 2–93 months) for PFS, with an OS rate of 89 % and 12 % for relapses.

### Eligibility criteria

The eligibility criteria were histologically proven BC, BM and blood samples obtained at the time of primary diagnosis and after neoadjuvant systemic therapy, no severe uncontrolled comorbidities or medical conditions, and no further malignancies at present or in the patient history.

Indications for NACT were: study participation for patients if comparable postoperative chemotherapy was indicated, patients with inflammatory BC, large operable BC primarily requiring mastectomy and adjuvant chemotherapy with the goal of breast conservation, such as patients with nondifferentiated or poorly differentiated tumors (G3) [47].

Neoadjuvant systemic therapy was performed according to guideline-based therapeutic regimens, including chemotherapy with anthracyclines, cyclophosphamides, 5-fluorouracil, and taxanes. In addition, patients with HER2-positive tumors were treated with HER2-targeted therapy (trastuzumab or lapatinib). Seven patients were treated with vascular endothelial growth factor targeted therapy with bevacizumab. Patients were included in clinical NACT trials and treated accordingly (e.g., the LAPADO, NeoALLTO, and GeparQuinto studies [48–50]). After completing NACT and surgery, patients were treated according to guidelines, including radiation, antihormonal therapy in those with hormone-responsive tumors (tamoxifen or an aromatase inhibitor), and trastuzumab therapy was completed for at least 1 year in patients with HER2 positivity [51, 52]. Additional oral clodronate therapy (2 × 520 mg per day for at least 2 years) was recommended in case of DTC positivity after therapy.

### Response criteria

Pathological response to therapy was defined according to the grading system of Sinn and colleagues [53] as pathological no response (regression according to Sinn 0 = no effect), pathological partial response [pPR; regression according to Sinn 1–3, where 1 = resorption and tumor sclerosis, 2 = minimal residual invasive tumor (<0.5 cm), and 3 = residual noninvasive tumor only; ductal carcinoma in situ (DCIS)], and pCR (defined as no evidence of residual invasive cancer and DCIS, both in breast and axilla; regression according to Sinn 4 = no tumor detectable).

### Collection and analysis of BM

Between 10 and 20 ml of BM was aspirated from the anterior iliac crests of 142 patients with primary BC before neoadjuvant systemic therapy during sentinel node biopsy or axillary lymph node dissection, as well as 165 patients during surgery of the tumor after NACT. Specimens were processed within 24 h. All specimens were obtained after written informed consent was provided, and they were collected using protocols approved by the clinical ethics committee of University Hospital Essen (05/2856). BM tumor cell isolation and detection were performed on the basis of recommendations for standardized tumor cell detection published by the German Consensus Group of Senology [54]. Details of the staining procedure (e.g., number of evaluated slides, controls, and cell detection) are described elsewhere [37, 55]. Briefly, BM cells were isolated from heparinized BM (5000 U/ml BM) by Ficoll-Hypaque density gradient centrifugation (density 1.077 g/mol; Pharmacia & Upjohn Diagnostics, Freiburg, Germany) at 400 × *g* for 30 minutes. Slides were analyzed for DTCs by immunocytochemistry using the pan-cytokeratin antibody A45-B/B3. Microscopic evaluation of the slides was carried out using the ARIOL system (Applied Imaging, Grand Rapids, MI, USA) according to the International Society of Hematotherapy and Graft Engineering evaluation criteria [56].

### Sampling of blood

Two 5 ml ethylenediaminetetraacetic acid blood samples were collected for isolation of CTCs before the application of therapeutic substances with an S-Monovette (Sarstedt AG & Co., Nümbrecht, Germany) and stored at 4 °C until further examination. The samples were processed immediately or at latest 4 h after blood withdrawal.

### Selection, detection, and evaluation of CTCs

Two 5 ml of blood before (*n* = 135 patients) and after (*n* = 133 patients) therapy were analyzed for CTCs with AdnaTest BreastCancer (QIAGEN Hannover GmbH) for the detection of transcripts of epithelial cell adhesion molecule (EpCAM); mucin 1, cell surface associated (MUC1); HER2; and β-actin. Expression of ER, PR, and ERCC1 was assessed in an additional reverse transcription polymerase chain reaction (RT-PCR) experiment. Establishment and validation of this assay are described in detail elsewhere [57, 58]. Briefly, all samples underwent immunomagnetic enrichment using the AdnaTest BreastCancerSelect (QIAGEN, Hannover GmbH). followed by RNA isolation and subsequent gene expression analysis by multiplex RT-PCR in separated tumor cells using the AdnaTest BreastCancerDetect (QIAGEN Hannover, GmbH). The primers generated fragments of the following sizes: GA 733-2, 395 bp; MUC1, 293 bp; HER2, 270 bp; PR, 270 bp; ER, 305 bp; ERCC1, 366 bp; and actin, 114 bp. Visualization of the PCR fragments was carried out with a 2100 Bioanalyzer using DNA 1000 LabChips (Agilent Technologies, Waldbronn, Germany) and the Expert software package (version B.02.03.SI307; Agilent Technologies).

### AdnaTest TumorStemCell and AdnaTest EMT

Both the AdnaTest TumorStemCell and AdnaTest EMT require the enrichment of CTCs from 5 ml of blood using AdnaTest BreastCancerSelect before a single-plex PCR assay to analyze ALDH1 and a multiplex PCR assay to analyze EMT markers and actin as an internal control. In total, the analysis of 148 healthy controls resulted in a specificity of 97 % and a sensitivity of 96 % for this test procedure, which is comparable to our previously published data in smaller cohorts [33, 34]. The primers generate fragments of the following sizes: ALDH1, 165 bp; AKT2, 306 bp; Twist-related protein 1 (TWIST1), 203 bp; phosphoinositide 3-kinase alpha (PI3Kα), 595 bp; and β-actin, 119 bp.

### Evaluation of data established for CTCs

The test result is considered positive if a PCR fragment of at least one tumor-associated transcript (MUC1, GA773-2, or HER2) is clearly detected. Using the software package for evaluation of the data on the Agilent 2100 Bioanalyzer, we found that peaks with a concentration >0.15 ng/μl were positive for the transcripts of GA733-2, MUC1, and HER2. Peaks with concentrations >0.60 ng/μl were positive for the ER transcript and >0.20 ng/μl were positive for the ERCC1 transcript. PR expression is considered positive when the transcript is detected without applying any cutoff. The cutoff values for the EMT markers and ALDH1 are 0.2 ng/μl for AKT2, 0.15 ng/μl for TWIST1, 0.25 ng/μl for PI3Kα, and 0.15 ng/μl for ALDH1.

### Immunohistochemical analysis of the primary tumor

For each of the 190 patients, the tumor type, TNM stage, and grade were assessed according to the World Health Organization classification of breast tumors [59] and the Sixth Edition of the TNM classification system [60]. ER and PR status were routinely determined by immunohistochemistry (IHC) in the pathology departments of each university hospital. The HercepTest score (Dako, Glostrup, Denmark) for the expression of HER2 was determined, and fluorescence in situ hybridization analysis was performed in cases of 2+ staining, as described elsewhere [61].

### Statistical analysis

The statistical analysis was performed using SAS® 9.2 software (SAS Institute, Cary, NC, USA). Summary statistics are presented as counts and percentages in the case of categorically scaled measures and as mean, median, standard deviation, and range in the case of continuously scaled variables. All cases with any available information were analyzed, and no imputation of missing information was foreseen.

The statistical analysis of relationships among the variables was performed in an exploratory way, starting with description by contingency tables (χ^2^ test or Fisher’s exact test for categorically scaled variables) or comparison of distributions (Mann-Whitney *U* test for continuously scaled variables). In parallel, univariable logistic regression models regarding binary outcome (yes or no) by factor were analyzed. Univariable Cox proportional hazards models were applied to investigate the influence of possible influencing variables on OS and PFS. In case of significant findings, Kaplan-Meier analyses were performed to create survival curves. The resulting odds ratio (OR) and hazard ratio (HR) are reported along with their 95 % confidence interval (CI) and *p* values. An α level of 0.05 was used, whereby an adjustment for multiple testing was not foreseen. Because of the limited sample size of patients with available cell count information and missing significant effects for cell counts on outcome measures in univariable analyses, a multivariable analysis was not performed.

## Results

### Patient characteristics

Clinical data are shown in detail in Table 1. The exact numbers of patients who had the different tests before and after NACT are shown in Additional file 1: Fig. S1. A total of 190 patients were included in the study. The median age of the patients was 51 years (range 18–84 years), and most women were either premenopausal (*n* = 87) or postmenopausal (*n* = 79). The predominant histologic subtype was invasive ductal carcinoma (*n* = 140), and most patients had grade II (*n* = 85) and grade III (*n* = 88) tumors. Tumor size before therapy was cT1a–cT1c in 46 patients (24 %), cT2 in 111 patients (58 %), and above cT2 in 29 patients (15 %). After therapy, tumor size was available in 177 patients, resulting in ypT0 tumors in 26 %, ypT1a–ypT1c tumors in 40 %, and ypT2 tumors in 27 % of cases. Twelve patients had tumors above ypT2. Before therapy, 94 patients were classified as node-negative. All other patients were cN1 (*n* = 83) and cN2 or cN3 (*n* = 10). After therapy, nodal status was available in 180 patients, resulting in 116 (64 %) node-negative and 64 (37 %) node-positive patients. ER positivity was observed in in 69 % (*n* = 131) and PR positivity in 61 % (*n* = 116) of the tumors. In 29 % (*n* = 56) of the cases, HER2 was overexpressed. Classifying tumors in subtypes on the basis of their receptor status, 51 % (*n* = 97) of the tumors were ER- and/or PR-positive and HER2-negative, 19 % (*n* = 36) were triple-negative (ER−/PR−/ HER2−), and 8 % (*n* = 15) of the tumors expressed only HER2 (ER−/PR−/HER2+). Response to therapy could be evaluated in 176 patients, resulting in ratios of 93 % responders (21 % complete response, 72 % partial response) and 7 % nonresponders.

### Detection of DTCs, CTCs, and slCTCs before and after NACT

DTCs were found in 38 (27 %) of 142 patients before and in 33 (20 %) of 165 patients after therapy (Table 1). In 118 patients, DTC status could be evaluated before and after NACT, resulting in positivity rates of 26 % before (31 of 118 patients) and 19 % (22 of 118 patients) after therapy. In 73 patients, no DTCs were detected at any time point, while 8 patients had persisting DTCs. Twenty-three of the thirty-one DTC-positive patients before NACT turned negative after chemotherapy, and fourteen patients with a negative DTC status before therapy had a positive DTC status after chemotherapy (Table 2).

**Table 2:** Paired analysis of tumor cells before and after NACT

	Tumor cells after NACT		
Tumor cells before NACT	Negative	Positive	Total
Total	96	22 (19 %)	118
DTC-negative	73	14	87
DTC-positive	23	8	31 (26 %)
Total	82	10 (11 %)	92
CTC-negative	58	9	67
CTC-positive	24	1	25 (27 %)
Total	39	9 (19 %)	48
slCTC-negative	18	1	19
slCTc-positive	21	8	29 (60 %)

*CTC* circulating tumor cell, *DTC* disseminated tumor cell, *NACT* neoadjuvant chemotherapy

**Fig. 1:**
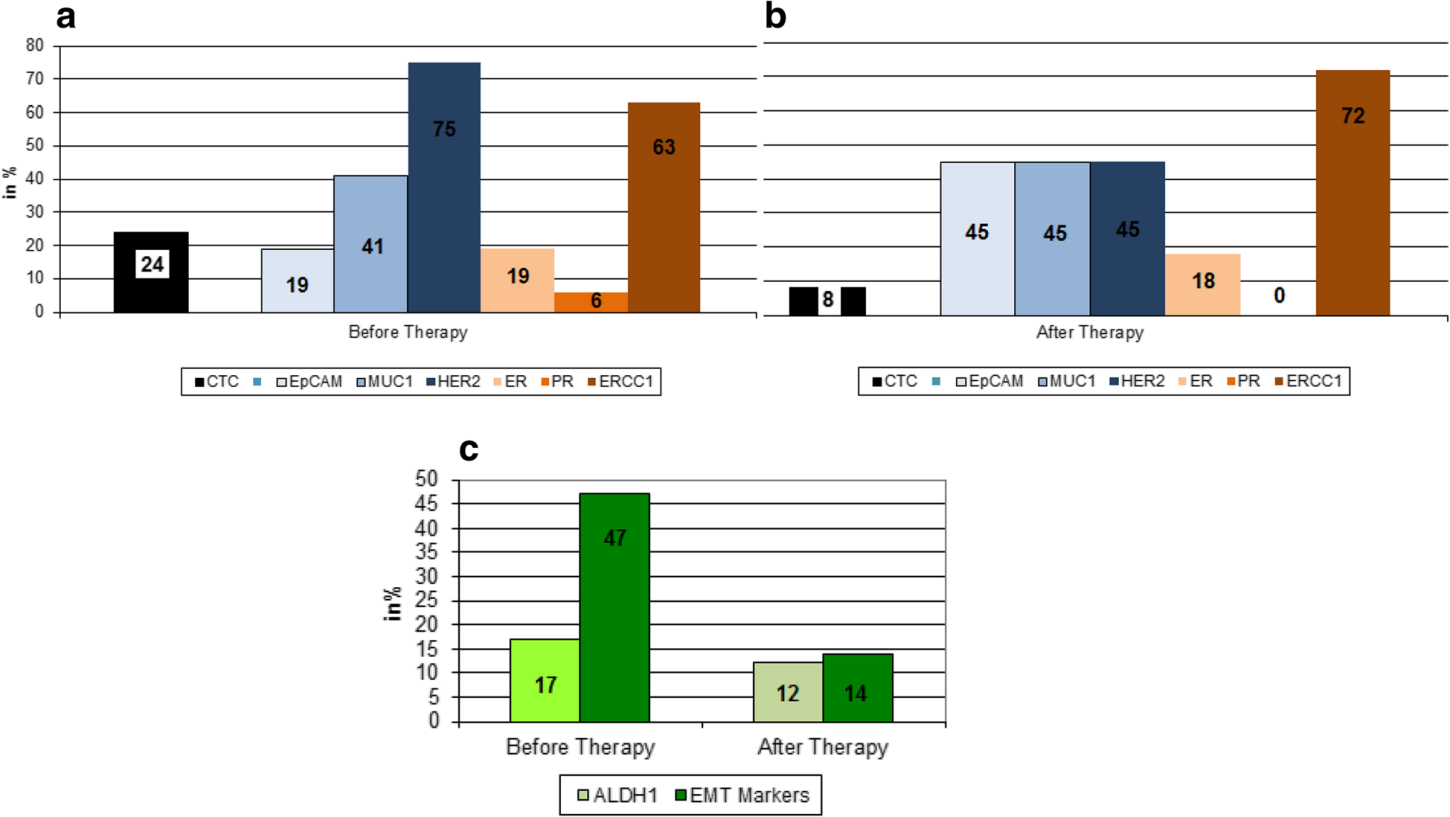
Comparison of expression profiles of circulating tumor cells (CTCs) before and after neoadjuvant chemotherapy (NACT). The expression rates of the different transcripts analyzed on CTCs are shown (**a**) before and (**b**) after NACT. **c** The presence of stem cell–like circulating tumor cell (slCTCs) is shown, including aldehyde dehydrogenase 1 (ALDH1)-positive cells and CTCs in epithelial–mesenchymal transition (EMT) before and after NACT. The identification of EMT markers was considered positive if at least one marker (phosphoinositide 3-kinase, AKT2, Twist-related protein 1) was detected in the sample. *EpCAM* epithelial cell adhesion molecule, *ERCC1* excision repair cross-complementing rodent repair deficiency, complementation group 1, *HER2* human epidermal growth factor receptor 2, *MUC1* mucin 1, cell surface associated

The detailed analysis for the evaluation of CTCs is illustrated in Fig. 1. Before therapy, CTCs were detected in 32 (24 %) of 135 of the patients expressing EpCAM (19 %), MUC-1 (41 %), HER2 (75 %), ER (19 %), PR (6 %), and ERCC1 (63 %). After therapy, 11 (8 %) of 133 of the patients were still positive for CTCs expressing EpCAM, MUC1, and HER2 (each 45 %); ER (18 %); PR (0 %); and ERCC1 (72 %). slCTCs were detected in 46 (51 %) of 91 of the patients before and in 18 (20 %) of 90 of the patients after therapy. Further, 47 % of the patients were positive for at least one of the EMT markers before and 14 % after therapy. For ALDH1, the values were 17 % and 12 %, respectively. Interestingly, after therapy, 50 % of the ERCC1-positive CTCs were also positive for ALDH1 and for at least one EMT marker.

Before therapy, 59 (43 %) of 136 patients were positive for DTCs and/or CTCs, 74 (69 %) of 107 were positive for DTCs and/or slCTCs, and 57 (62 %) of 92 were positive for CTCs and/or slCTCs. After therapy, the corresponding values were 34 % (44 of 140 patients), 47 % (48 of 103 patients), and 28 % (25 of 89 patients), respectively. The presence of CTCs before (OR 4.200, 95 % CI 1.549–11.389, *p* = 0.005) and after therapy (OR 0.688, 95 % CI 0.177–2.676, *p* = 0.05) was significantly associated with the presence of slCTCs (Tables 3 and 4).

**Table 3:** Correlation between clinical data and DTCs, CTCs, and slCTCs before therapy

	Total	DTC-positive, *n* (%)	*p* Value	Total	CTC-positive, *n* (%)	*p* Value	Total	slCTC-positive (%)	*p* Value
Tumor size	139	37 (27)		132	31 (23)		89	44 (49)	
cT1	32	5 (16)	0.27	32	10 (31)	0.26	26	15 (58)	0.56
cT2	86	25 (29)		81	19 (23)		53	25 (47)	
Above cT2	21	7 (33)		19	2 (11)		10	4 (40)	
Nodal status	141	38 (27)		134	32 (24)		90	45 (50)	
cN0	77	28 (36)	0.03	74	21 (28)	0.62	46	24 (52)	0.44
cN1	56	9 (16)		53	11 (21)		39	20 (51)	
cN2, cN3	8	1 (13)		7	0 (0)		5	1 (20)	
Histology	140	37 (26)		133	32 (24)		90	45 (50)	
Ductal	106	27 (25)	0.046	99	22 (22)	0.70	67	33 (49)	0.80
Lobular	16	8 (50)		17	5 (29)		12	7 (58)	
Others	18	2 (11)		17	5 (29)		11	5 (45)	
Grade	139	38 (27)		132	32 (24)		89	44 (49)	
I	9	2 (22)	0.87	12	0 (0)	0.31	5	3 (60)	
II	65	19 (29)		59	12 (20)		40	17 (43)	0.49
III	65	17 (26)		61	20 (33)		44	24 (55)	
ER status	141	38 (27)		134	32 (24)		90	45 (50)	
Negative	43	8 (17)	0.14	41	8 (20)	0.43	24	11 (46)	0.63
Positive	98	30 (31)		93	24 (26)		66	34 (52)	
PR status	141	38 (27)		134	32 (24)		90	45 (50)	
Negative	60	14 (23)	0.41	56	13 (23)	0.88	39	19 (49)	0.83
Positive	81	24 (30)		78	19 (24)		51	26 (51)	

*CTC* circulating tumor cell, *DTC* disseminated tumor cell, *ER* estrogen receptor, *PR* progesterone receptor, *slCTC* stem cell–like circulating tumor cell

**Table 4:** Correlation between clinical data and DTCs, CTCs, and slCTCs after therapy

	Total	DTC-positive, *n* %	*p* Value	Total	CTC-positive, *n* %	*p* Value	Total	slCTC-positive, *n* %	*p* Value
Tumor size	163	33 (20)		131	11 (8)		88	18 (20)	
ypTis, ypT0	43	12 (28)	0.25	36	3 (8)	0.86	21	3 (14)	0.50
ypT1a	15	2 (13)		13	0 (0)		12	1 (8)	
ypT1b, ypT1c	52	6 (12)		40	4 (10)		26	5 (19)	
ypT2	41	11 (27)		31	2 (6)		23	7 (30)	
Above ypT2	12	2 (17)		11	2 (18)		6	2 (33)	
Nodal status	163	33 (20)		131	11 (8)		88	18 (20)	
yN0	103	26 (25)	0.13	83	6 (7)	0.17	56	11 (20)	0.73
yN1	44	5 (11)		35	2 (6)		22	4 (18)	
yN2, yN3	46	2 (13)		13	3 (23)		10	3 (30)	
Histology	163	33 (20)		131	11 (8)		89	18 (20)	
Ductal	122	28 (23)	1.00	94	9 (10)	0.75	66	14 (21)	0.83
Lobular	21	5 (24)		19	1 (5)		9	2 (22)	
Other	20	0 (0)		18	1 (6)		14	2 (14)	
Grade	162	32 (20)		130	11 (8)		88	18 (20)	
I	11	2 (18)	0.95	9	2 (22)	0.35	3	1 (33)	0.85
II	72	15 (21)		55	4 (7)		39	8 (21)	
III	79	15 (19)		66	5 (8)		46	9 (20)	
ER status	165	33 (20)		133	11 (8)		90	18 (20)	
Negative	52	14 (27)	0.13	43	1 (2)	0.12	30	3 (10)	0.11
Positive	113	19 (17)		90	10 (11)		60	15 (25)	
PR status	165	33 (20)		133	11 (8)		90	18 (20)	
Negative	62	17 (27)	0.07	49	2 (4)	0.20	35	5 (14)	0.28
Positive	103	16 (16)		84	9 (11)		55	13 (24)	

*CTC* circulating tumor cell, *DTC* disseminated tumor cell, *ER* estrogen receptor, *PR* progesterone receptor, *slCTC* stem cell–like circulating tumor cell

In 92 patients, CTC status could be evaluated before and after NACT. We found positivity rates of 27 % before (25/92 patients) and 11 % (10/92 patients) after therapy. Interestingly, CTCs were eradicated in 24 of 25 CTC-positive patients before therapy, while only 1 patient had persisting CTCs and 9 (13 %) of 67 patients had a switch from negative to positive CTC status (Table 2). In addition, the switch from CTC-positive before to CTC-negative after NACT appeared more often in post- and perimenopausal women than in premenopausal women (*p* = 0.0499).

slCTC status before and after NACT was available in 48 patients, with positivity rates of 60 % (29 of 48 patients) before and 19 % (9 of 48 patients) after therapy. Eight patients had persisting slCTCs, while eighteen patients were negative/negative, 21 patients were positive/negative, and only one patient was negative/positive (Table 2). Furthermore, the eradication of slCTCs was rarer the bigger the tumor size (*p* = 0.044).

### Correlation of tumor cells before and after NACT with clinical characteristics

The correlation between the presence of tumor cells and clinical characteristics before and after therapy is shown in Tables 2 and 3. Whereas no significant associations with clinical parameters were found for CTCs and slCTCs before and after therapy, DTCs were significantly associated with nodal status (OR 0.335, 95 % CI 0.143–0.785, *p* = 0.03 for N0 vs N1) and histology (OR 2.926, 95 % CI 1.001–8.556, *p* = 0.046 for ductal vs lobular; OR 0.125, 95 % CI 0.021–0.732, *p* = 0.023 for lobular vs. other) before therapy and the IHC subtype (OR 3.954, 95 % CI 1.564–9.997, *p* = 0.02 for triple-negative vs. ER and/or PR-positive but HER2-negative). Although no significant correlations were observed for response and slCTCs before therapy, logistic regression identified a significant relationship between slCTCs and the group of complete responders vs. no remission (OR 0.091, 95 % CI 0.009–0.880, *p* = 0.04).

### Survival analysis

**Fig. 2:**
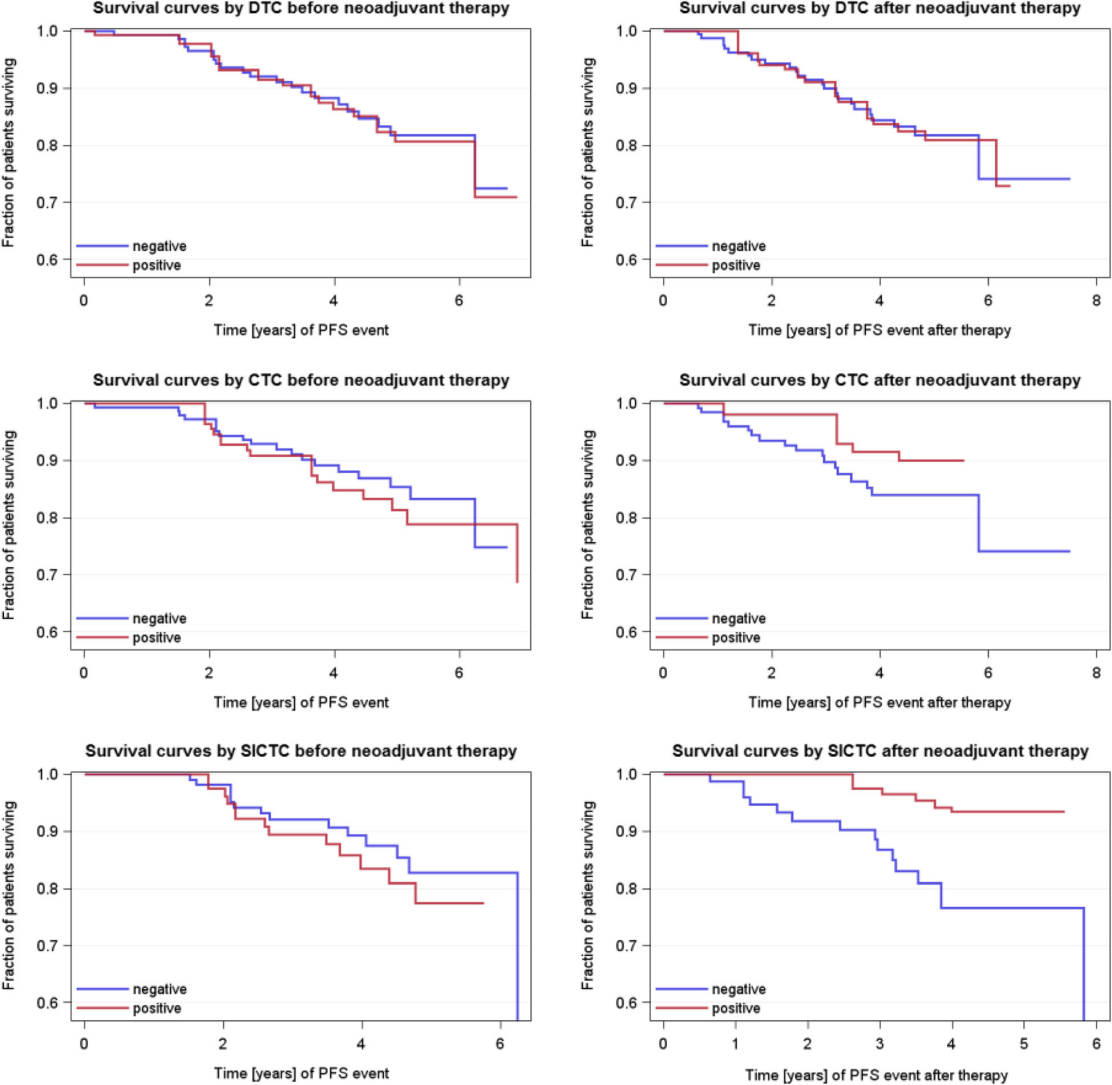
Prognostic capability of DTCs, CTCs, and slCTCs before and after NACT with regard to PFS. Kaplan-Meier curves were drawn to compare PFS with regard to CTCs/slCTCs in blood and DTCs in BM before and after NACT. No significant associations were found with regard to PFS for every cell type tested (for DTCs, HR 1.065, 95 % CI 0.411–2.763, *p* = 0.9190 before therapy; HR 1.053, 95 % CI 0.393–2.821, *p* = 0.8969 after therapy; for CTCs, HR 1.298, 95 % CI 0.466–3.620, *p* = 0.6179 before NACT; HR 0.607, 95 % CI 0.080–4.582, *p* = 0.6285 after NACT; and for slCTCs, HR 1.346, 95 % CI 0.450–4.032, *p* = 0.5950 before therapy; HR 0.254, 95 % CI 0.033–1.942, *p* = 0.1865 after therapy). In the univariable Cox regression model estimated survival curves, *blue line* = DTC/CTC/slCTC-negative patients and *red line* = DTC/CTC/slCTC-positive patients. *BM* bone marrow, *CI* confidence interval, *CTC* circulating tumor cell, *DTC* disseminated tumor cell, *HR* hazard ratio, *NACT* neoadjuvant chemotherapy, *PFS* progression-free survival, *slCTC* stem cell–like circulating tumor cell

**Fig. 3:**
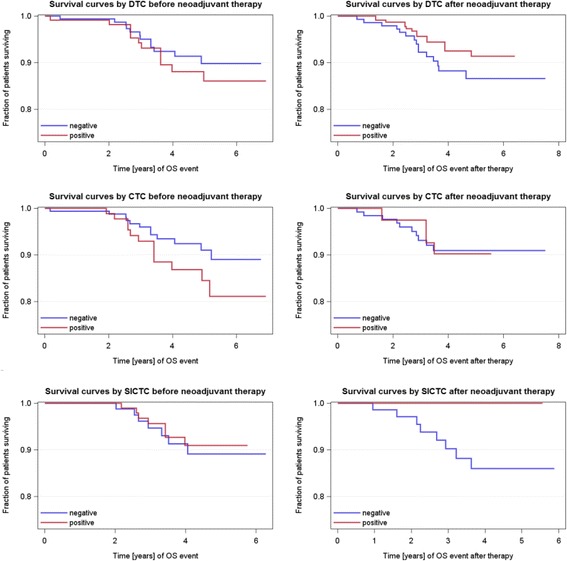
Prognostic capability of DTCs, CTCs, and slCTCs before and after NACT with regard to OS. Estimated survival curves adjusted for cell type status were drawn to compare OS with regard to CTCs/slCTCs in blood and DTCs in BM before and after NACT. No significant associations were found with regard to OS for every cell type tested [for DTCs, HR 1.404, 95 % CI 0.422–4.664, *p* = 0.5800 before therapy; HR 0.628, 95 % CI 0.142–2.786, *p* = 0.5406 after therapy; for CTCs, HR 1.795, 95 % CI 0.538–5.987, *p* = 0.3414 before NACT; HR 1.083, 95 % CI 0.137–8.550, *p* = 0.9400 after NACT; for slCTCs, HR 0.830, 95 % CI 0.186–3.710, *p* = 0.8068 before and after therapy; and not applicable (no events for slCTC-positive patients)]. *BM* bone marrow, *CI* confidence interval, *CTC* circulating tumor cell, *DTC* disseminated tumor cell, *HR* hazard ratio, *NACT* neoadjuvant chemotherapy, *OS* overall survival, *slCTC* stem cell–like circulating tumor cell

As shown in Table 1, the median follow-up time for PFS was 52 months (range 2–93 months), and for OS it was 54 months (range 2–93 months). The OS (not BC-specific) rate was 89 %. Relapses occurred in 12 % of cases, 3 patients had local recurrence, 16 patients had a distant recurrence, and 3 patients had both local and distant recurrence. Univariable Cox regression analysis revealed age (*p* = 0.0065), tumor size before (*p* = 0.0473), nodal status (*p* = 0.0137) after NACT, and response to NACT (*p* = 0.0136) were also significantly correlated with PFS, whereas age (*p* = 0.0162) and nodal status after NACT (*p* = 0.0243) were significantly associated with OS (Table 5). No significant correlations were found for DTCs or any CTCs before and after therapy with regard to PFS and OS (Figs. 2 and 3). In addition, all combinations tested between the presence of DTCs and/or any CTCs, as well as their change before and after therapy, and PFS and OS did not show any significant results (data not shown).

**Table 5:** Univariable Cox regression analysis of survival

	PFS		OS	
	HR	*p* Value	HR	*p* Value
Age		0.0065		0.0162
Tumor size before therapy				
cT1 vs. cT2	7.621 (1.024–56.707)	0.0473	n.s.	
Nodal status after therapy				
yNo vs. yN2, yN3	3.377 (1.283–8.890)	0.0137	4.104 (1.201–14.027)	0.0243
Response to NACT				
Partial response vs. no response	3.107 (1.157–8.344)	0.0245	n.a.	
Complete response vs. no response	16.974 (1.971–145.174)	0.0099	n.s.	

*HR* hazard ratio, *NACT* neoadjuvant chemotherapy, *OS* overall survival, *PFS* progression-free survival; *n.a.* not applicable,* n.s.* not significant.

## Discussion

Primary systemic NACT in BC is considered to convert inoperable tumors to operable primary tumors and is undertaken to improve surgical options. Furthermore, NACT reflects the tumor response to treatment and allows a more individual therapeutic concept. Response to NACT has been associated with improved PFS and OS. The OS rate in our sample was 89 %, as compared with an overall OS rate of 87 % in Germany [62]. Taking into account that we exclusively had a high-risk patient cohort undergoing NACT, we documented a better OS than the overall OS rate of patients with BC in Germany, containing high- as well as low-risk patients. Furthermore, we showed a significant correlation of response to NACT with regard to PFS when patients with pCR or pPR were compared with nonresponders. For OS, no significant associations were found, probably due to the fact that the observation period was still too short. However, some patients experienced relapse, even those having achieved a pCR, as recently published [63]. This indicates that the disease is able to persist in secondary organs such as the BM, which might have spread tumor cells into the circulation. We demonstrate that, although CTCs were eradicated more effectively than DTCs, CTCs detected after treatment seemed to be associated with tumor cells showing tumor stem cell characteristics as well as resistant tumor cell populations, which might indicate worse outcome in the future. These findings underline our assumption that CTCs, probably circulating from reservoirs in the lung or liver, might be a high-risk indicator for already ongoing metastasis not limited to bone metastasis.

Thus, these patients might benefit from additional second-line treatment protocols for the eradication of minimal residual disease. However, after a median follow-up time of nearly 5 years, no significant correlations were found for DTCs, CTCs, and slCTCs as well as for their changes before and after therapy with regard to PFS and OS.

### Disseminated tumor cells

DTCs have been analyzed mainly after NACT, with a detection rate of 40–50 % but demonstrating that 30 % of the detected cells were apoptotic [21, 64]. In only one study have researchers analyzed these cells before and after NACT, showing positivity rates of 21 % and 16 %, respectively, which is quite in accord with our data showing 27 % positivity before and 19 % after NACT, respectively [11]. Interestingly, 14 of 87 patients with a negative DTC status before therapy switched to a positive DTC status after chemotherapy. We can only speculate and hypothesize that these patients probably were DTC-positive before therapy and that DTCs probably had undergone EMT and lost their epithelial character, which allowed them to travel to metastatic sites without being affected by conventional treatment [65].

Furthermore, DTCs in our patients were significantly associated with nodal status before and after NACT as well as with the histology- and IHC-determined subtype before NACT, while no significant correlations could be documented for PFS and OS, which is in contrast to the results obtained in the above-mentioned studies. The fact that DTCs persist after treatment and are associated with worse outcome has already been described and is explained by a clinically significant biological heterogeneity, most likely due to phenotypical changes, EMT and stem cell characteristics, and altered genomic characteristics between DTCs, CTCs, and the primary tumor [9–11, 32, 35, 66–70]. One treatment option for DTCs has been demonstrated by Naume et al., who administered six cycles of docetaxel as secondary treatment in DTC-positive patients after first-line therapy with fluorouracil, epirubicin, and cyclophosphamide, resulting in better survival of these patients [71]. We did not further characterize DTCs; thus, we can only speculate that residual cells might have stem cell characteristics in some patients. However, we did not see any negative prognostic effect of DTCs before and/or after NACT with regard to PFS and OS after a median follow-up of nearly 5 years, which is in accord with our other published adjuvant BC studies, where we demonstrated that the intake of clodronate in case of DTC positivity was able to eradicate DTCs even years after the first diagnosis [26, 72]. Thus, clodronate intake was also offered to DTC-positive patients in this study. However, it is still unknown whether bisphosphonates are also able to eradicate stem cell–like DTCs present among DTCs in the BM. The fact that stem cell–like and EMT-like cells, probably circulating from reservoirs other than the BM (e.g. liver, lung), were present in blood samples before and after therapy makes the hypothesis that DTCs with stem cell–like characteristics are eradicated by bisphosphonates more or less unlikely.

### Circulating tumor cells

CTCs have been suggested to be potential surrogate markers for minimal residual disease, the precursor of metastatic disease. There is increasing evidence that CTCs could be a strong predictive biomarker of response to NACT because BM is too invasive and painful for monitoring purposes. Unfortunately, a recently published meta-analysis confirmed that, although CTCs before NACT significantly correlated with reduced PFS in some studies, the change (decrease or increase) in CTC numbers during NACT in patients with locally advanced BC was not associated with pCR, and a decrease in CTC counts after NACT did not indicate that patients had an improved response [44].

The main problem with using CTCs as a so-called liquid biopsy is the fact that, at present, there is no standard definition for the identification of CTCs [73]. Currently, the CELLSEARCH system, based on immunomagnetic EpCAM capturing, is the only system for CTC enumeration in BC approved by the U.S. Food and Drug Administration [7]. However, despite the prognostic impact of CTC counts in BC, it has been shown that this procedure is not able to detect the entire, highly heterogeneous population of CTCs, including slCTCs and CTCs in EMT [74, 75]. Despite the prognostic impact of CTC counts, it is indispensable to characterize these cells, which might complement these studies by improving the overall detection rate as well as sensitivity and thus permit the assessment of genomic markers in CTCs of patients with BC, as recently published [45].

Although we and others, by using molecular methods, have already characterized the heterogeneous CTC populations in primary BC before adjuvant therapy [7, 33, 37, 38, 76, 77], in few studies have researchers evaluated the presence, not the characteristics, of CTCs after adjuvant therapy, resulting in a positivity rate of 22–34 % [17, 20, 78]. Furthermore, in the neoadjuvant setting, the number of circulating epithelial cells was mostly used for monitoring the effect of chemotherapy after every cycle of treatment [23, 24]. To the best of our knowledge, our present study is the first in which CTCs have been characterized so comprehensively before and after NACT. In contrast to DTCs, most of the CTCs before therapy, present in about 24 % of the patients and reflecting the heterogeneous CTC population, were eliminated by the given therapy. Although we cannot definitively prove that all residual CTCs were stem cell–like and EMT-like, molecular marker expression might allow characterization of them as stem cell–like. In addition, 72 % of the residual cells were characterized as ERCC1-positive, indicating therapy-resistant tumor cell populations. Interestingly, seven of these eight patients had been treated with taxanes or anthracyclines, which were shown to eradicate DTCs in a Norwegian study [13]. The fact that ERCC1-positive CTCs were present after these therapies might indicate that these cells survived treatment. This knowledge might help clinicians to decide more precisely about further secondary treatment options in the future. However, CTCs and slCTCs before as well as after therapy did not significantly correlate with decreased PFS or OS, as described in the above-mentioned studies. On one hand, follow-up time might have been too short to see an effect on outcome; on the other hand, factors determining if single tumor cells form metastasis have not been identified yet. In this regard, it has been demonstrated that CTCs can be detected after more than 20 years in patients with BC without any sign of relapse [79].

### Secondary treatment options

Tumor stem cells are well known to be resistant to various chemotherapeutic agents and radiotherapy [80]. In this context, Creighton et al. reported supportive evidence that the residual breast tumor tissue cell populations surviving after letrozole or docetaxel were enriched for subpopulations of cells with both tumor-initiating and mesenchymal features [41]. Thus, additional therapeutic strategies are urgently needed to prevent relapse of the disease. In this regard, signaling pathways that maintain cancer stem cells are attractive targets for these therapies. One example is everolimus (RAD001), an oral inhibitor of mammalian target of rapamycin acting downstream of the PI3K/AKT pathway, which was shown to have effective inhibitory effects on cancer stem cells in vitro and in vivo, and combination treatment with RAD001 and docetaxel or trastuzumab has been reported to be effective in refractory metastatic BC [81]. Li et al. demonstrated that remaining tumorigenic cells after chemotherapy had unique properties of enhanced self-renewal as demonstrated by formation of mammospheres and increased propensity for tumor formation. In addition, lapatinib did not lead to an increase in these tumorigenic cells; thus, in combination with conventional therapy, specific pathway inhibitors may provide a therapeutic strategy for eliminating these cells to decrease recurrence and improve long-term survival [82]. Concerning HER2, a recently published study suggested that the clinical efficacy of adjuvant trastuzumab may relate to the ability of this agent to target the cancer stem cell population in a process that does not require HER2 gene amplification [83]. These results are partly comparable with ours because we observed clearance of slCTCs after NACT in some patients who received trastuzumab, lapatinib, or bevacizumab, whereas patients who did not receive these combinations had slCTCs left after therapy (data not shown). These observations have to be interpreted with caution, and more patients have to be followed to prove whether this observation holds true. Further promising agents that are thought to attack BC stem cells are salinomycin, where treatment resulted in the loss of expression of BC stem cells [84], and a new synthetic curcumin analogue against ALDH1 and glycogen synthase kinase-3β [85]. In the future, targeting the tumor microenvironment, such as by interrupting the immune cells (e.g., myeloid-derived suppressor cells) and cytokines [e.g., interleukin (IL)-6, IL-8] as well as the immune checkpoints [programmed cell death protein 1/programmed cell death ligand 1 (PD1/PDL1)], may provide additional new tools for immunological targeting of cancer stem cells [86].

## Conclusions

CTCs were eradicated effectively by NACT; however, CTCs detected after treatment seemed to be associated with tumor cells showing tumor stem cell characteristics as well as resistant tumor cell populations, which might indicate worse outcomes in the future. These patients might benefit from additional second-line treatment protocols that attack BC stem cells. In contrast, DTCs were not targeted effectively by NACT and were still present in about 20 % of patients after therapy. Nevertheless, DTCs were not associated with worse outcomes, probably due to clodronate intake as a secondary treatment option for the eradication of DTCs.

## Additional file

Additional file 1:
**Exact numbers of patients who had the different tests before and after NACT.**(PDF 34 kb)

## Abbreviations

ALDH1: aldehyde dehydrogenase 1
BC: breast cancer
BM: bone marrow
CTC: circulating tumor cell
DCIS: ductal carcinoma in situ
DTC: disseminated tumor cell
EMT: epithelial–mesenchymal transition
EpCAM: epithelial cell adhesion molecule
ER: estrogen receptor
ERCC1: excision repair cross-complementing rodent repair deficiency, complementation group 1
HER2: human epidermal growth factor receptor 2
HR: hazard ratio
IHC: immunhistochemistry
IL: interleukin
MUC1: mucin 1, cell surface associated
NACT: neoadjuvant chemotherapy
OR: odds ratio
OS: overall survival
pCR: pathological complete response
PFS: progression-free survival
PI3K: phosphoinositide 3-kinase
pNR: pathological no response
pPR: pathological partial response
PR: progesterone receptor
RT-PCR: reverse transcription polymerase chain reaction
slCTC: stem cell–like circulating tumor cell
TNM: tumor, node, metastasis stage
TWIST1: Twist-related protein 1
ZOL: zoledronic acid
