# Supplementary material for: Does primary neoadjuvant systemic therapy eradicate minimal residual disease? Analysis of disseminated and circulating tumor cells before and after therapy
Source: Breast Cancer Res. 2016 Feb 12;18:20. doi: 10.1186/s13058-016-0679-3 (PMC4751719; doi:10.1186/s13058-016-0679-3)
Supplement: Additional file 1: — Exact numbers of patients who had the different tests before and after NACT.(PDF 34 kb) [file 13058_2016_679_MOESM1_ESM.pdf]

included in the study	190

	CTC				DTC				OR (CTC, DTC)			
Exist before NATC	y	y	n	n	y	y	n	n	y	y	n	n
Exist after NATC	y	n	y	n	y	n	y	n	y	n	y	n
	92	43	1	14	118	27	47	1	97	39	43	11

y = yes / done
n = no / not done

sSICTC			
y	y	n	n
y	n	y	n

48	42	43	57
